# Are Unfamiliar Neighbours Considered to Be Dear-Enemies?

**DOI:** 10.1371/journal.pone.0012428

**Published:** 2010-08-26

**Authors:** Elodie Briefer, Fanny Rybak, Thierry Aubin

**Affiliations:** CNPS, CNRS-UMR 8195, Université Paris Sud, Orsay, France; University of Utah, United States of America

## Abstract

**Background:**

Discriminating threatening individuals from non-threatening ones allow territory owners to modulate their territorial responses according to the threat posed by each intruder. This ability reduces costs associated with territorial defence. Reduced aggression towards familiar adjacent neighbours, termed the dear-enemy effect, has been shown in numerous species. An important question that has never been investigated is whether territory owners perceive distant neighbours established in the same group as strangers because of their unfamiliarity, or as dear-enemies because of their group membership.

**Methodology/Principal Findings:**

To investigate this question, we played back to male skylarks (*Alauda arvensis*) songs of adjacent neighbours, distant neighbours established a few territories away in the same microdialect area and strangers. Additionally, we carried out a propagation experiment to investigate how far skylark songs are propagated in their natural habitat and we estimated repertoire similarity between adjacent neighbours, distant neighbours and strangers. We show that skylarks, in the field, respond less aggressively to songs of their distant and likely unfamiliar neighbours, as shown by the propagation experiment, compared to stranger songs. The song analysis revealed that individuals share a high amount of syllables and sequences with both their adjacent and distant neighbours, but only few syllables and no sequences with strangers.

**Conclusions:**

The observed reduction of aggression between distant neighbours thus probably results from their familiarity with the vocal group signature shared by all members of the neighbourhood. Therefore, in skylarks, dear-enemy-like relationships can be established between unfamiliar individuals who share a common acoustic code.

## Introduction

The ability to discriminate potential territory and/or mate usurpers from non-threatening individuals enables territory owners to modulate their territorial responses according to the threat posed by each intruder. In songbirds, males commonly hold adjacent territories forming neighbourhoods, within which they share whole songs or song components (microdialects, reviewed in [1]). A weak territorial reaction towards adjacent familiar individuals compared to unfamiliar individuals (“strangers”, “dear-enemy” effect [2]), has been observed in numerous songbirds (review [3]). Social relationships between males are likely to go beyond neighbours with whom territory boundaries are shared [4], but territorial reactions towards less familiar neighbours established some territories away in the same neighbourhood (hereafter “distant neighbours”) have never been investigated.

Theoretically, within a neighbourhood, distant and adjacent neighbours, which are already in possession of a suitable territory, present a similarly low level of threat for territory owners compared to strangers, which could potentially be floaters looking for a territory. Thus, birds may benefit from recognizing both categories of neighbours and from showing reduced territorial aggression towards them. As the probability of hearing songs produced by a given individual decreases with the emitter-receiver distance, birds are likely to be unfamiliar with their distant neighbours' songs, or a lot less familiar than with their adjacent neighbours' songs. An unexplored question is whether territory owners perceive distant neighbours as strangers because of the unfamiliarity with their songs, or as neighbours because of their group membership, signalled by particular shared song components. Recognition of familiar song components within unfamiliar songs has been studied in laboratory conditions (e.g. [5], [6]), but has rarely been explored in the field (but see [7], [8]). Here, we investigated vocal distant neighbour recognition in a songbird with a large repertoire (average repertoire size per male  = 340 syllables [8]) and a complex song, the skylark *Alauda arvensis*, in its natural environment. To study this process, we considered for the first time the three following related problems: 1) how territory owners respond to songs of their adjacent neighbours, of their distant neighbours and of strangers; 2) how far skylark songs are propagated in their natural habitat and 3) what is the degree of repertoire sharing between individuals.

**Figure 1:**
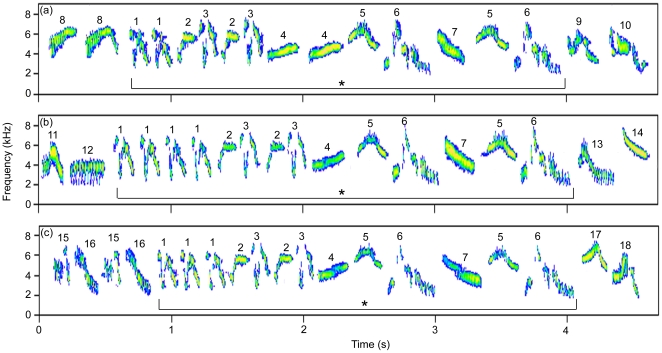
Sequence shared by members of the neighbourhood (microdialect). Spectrograms (FFT length, 256; frame, 100%; Hanning window) of song parts produced by an individual (ind a), his adjacent neighbour (ind b) and his distant neighbour (ind c) whose songs had been broadcast during the playback experiment, all including the same shared sequence (*). Syllables composing the shared sequence are labelled with numbers 1 to 7, and the other syllables with numbers 8 to 18.

During the breeding season, skylark pairs settle in territories gathered in distinct groups of neighbours spaced by few kilometres because of habitat heterogeneity. Within a neighbourhood, males defend stable and adjacent territories with conspicuous territorial behaviour [9]. Their long and continuous song is an obvious element of this behaviour. In this song, particular sequences of syllables are shared by all males of a group (microdialect [8], [10], Fig. 1). When boundaries between territories are stable, adjacent neighbours establish dear-enemy relationships, reacting weakly to each other's songs played from the shared territory boundary and aggressively to songs of strangers from other groups [8], [11].

We tested two alternative hypotheses by playing back songs of adjacent neighbours, distant neighbours established a few territories away in the same microdialect area and strangers:

1. A male's response to a given song depends on its familiarity with the singer. In this case, responses to a song will increase with the distance to the bird singing this song. We thus predict lowest responses to adjacent neighbour songs, intermediate responses to distant neighbour songs and high responses to stranger songs.
2. A male's response to a given song depends on the presence in this song of familiar song components shared by members of the neighbourhood. In this case, responses to a neighbour song will not be related to the distance to the bird singing this song. We expect similar responses to adjacent and distant neighbour songs, regardless of the distance to these neighbours, and higher responses to stranger songs.

Additionally, we carried out a propagation experiment to investigate how far skylark songs are propagated in an open landscape corresponding to skylark habitat. This allowed us to characterize the vocal active space of each individual in a group. We also analysed repertoire similarity between adjacent neighbours, distant neighbours and strangers to characterize the spreading of vocal sharing within a neighbourhood.

## Methods

### Study area, subjects and song recordings

We carried out our study during the 2008 breeding season, from March to May, in the fields surrounding the University of Paris South, France. Subjects were 17 males established in 2 groups of respectively 16 and 19 neighbours, separated by 5.8 km. Within a group, individuals were established in adjoining and stable territories of circa 1 ha. We estimated territory boundaries after careful observations of the birds' movements at different times of day and recorded GPS coordinates at the centre of each territory. Distances between two GPS coordinates where then calculated in metres with a calculator using a spherical earth assumption. We recorded several songs per individual between 0900 and 1200 hours Eastern Daylight Time using a Marantz PMD 690 numeric recorder (sampling rate: 48 kHz) connected to a Sennheiser ME 64 K6 omnidirectional microphone (frequency response: 30 Hz to 20 kHz ±1 dB) mounted on a Telinga Universal parabola (diameter: 50 cm). We then transferred song files to a computer and high-pass filtered (cut-off frequency: 1600 Hz) to remove background noise. We used Avisoft SASLab pro v.4.31 software [12], Goldwave v.5.11 [13] and Seewave [14] for the preparation of songs played back and for subsequent analyses.

### Playback Experiment

#### (a) Signals tested

**Figure 2:**
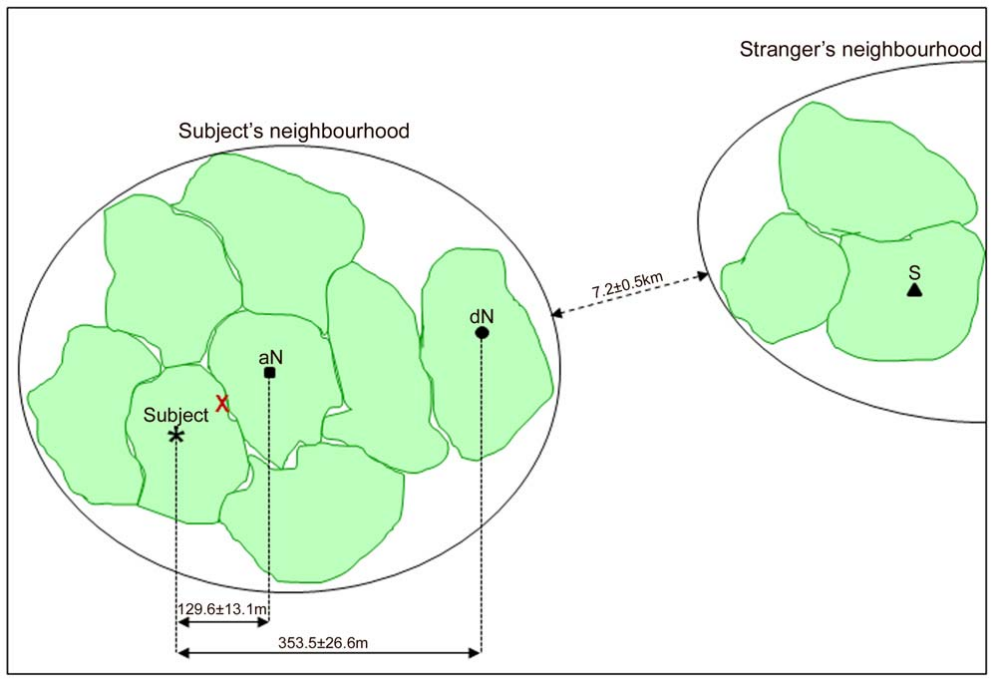
Playback experiment design. Schematic overview of the territories of a tested subject (*) and the adjacent Neighbour (aN, square), the distant Neighbour (dN, circle) and the Stranger (triangle) whose songs were played at the subject's territory boundary (X). Distances between the subject's territory and the territories of the adjacent and distant Neighbours, and between the subject's neighbourhood and the Stranger's neighbourhood are indicated (mean ± SE).

We tested each subject with three song treatments: an adjacent Neighbour (aN) song, i.e. a song produced by one of its adjacent neighbours (distance between the centre of the territory of a subject and its adjacent neighbour: mean ± SE  = 129.56±13.14 m, *n* = 17); a distant Neighbour (dN) song, i.e. a song produced by a neighbour established 2 to 3 territories, i. e. 230 to 570 m away, on the same side as the adjacent neighbour's territory (distance between the centre of the territory of a subject and its distant neighbour: mean ± SE  = 353.53±26.56 m, *n* = 17); and a Stranger (S) song, i.e. a song produced by a territory owner established in a distant group (mean ± SE distance between the group tested and the group of the stranger: 7.23±0.49 km, *n* = 7, Fig. 2). To avoid pseudo-replication [15], we prepared a different aN and dN song for each subject, and five different S songs for each group (10 S songs in total, recorded in 7 different groups), so that each S song was played back to a mean ± SE of 1.50±0.17 individuals. We adjusted all song stimuli to the same duration by taking the first 90 s of continuous song. We rescaled each song to match the root mean square (RMS) amplitude of the other stimuli.

#### (b) Playback procedure

We played back songs with a Marantz-PMD 690 digital recorder connected via a 20 m cable to a 10 W Megavox-6000 loudspeaker (frequency response: 400 Hz to 10 kHz ±3 dB), at the intensity estimated to be normal for the birds (mean ± SE: 90.8±0.8 dB_SPL_ measured at 1 m from the loudspeaker with a Brüel & Kjaer 2235 sound level meter, linear setting). We positioned the loudspeaker on the ground at approximately 5 m from the boundary within the subject's territory, on the side shared with the adjacent neighbour whose song was used as a stimulus. The experimenter stood at 20 m from the loudspeaker. The 3 song treatments (aN, dN and S songs) were broadcast randomly on the same day to each subject, separated by at least 5 min delay. This time interval allowed the birds to return to a normal activity. We initiated the playback when the subject was standing on the ground inside its territory at more than 10 m from the loudspeaker.

#### (c) Responses measured and statistical analyses

For each trial, we scored the response of the bird during 180 s, corresponding to the broadcast of 90 s of continuous song and 90 s of post-playback observation. We recorded 4 measures of response to assess the effect of the different song treatments (Table 1). Composite response measures were derived from a principal components analysis (PCA, correlation matrix) of the 4 original response measures, which are likely to be correlated [15]. We retained the first two principal components of the PCA (PC1 and PC2), which had eigenvalues greater than 1 (Kaiser's criterion), and tested and confirmed their scores for normality (Kolmogorov-Smirnov test). We compared PC1 scores corresponding to the different song treatments (aN, dN and S songs) using a factorial ANOVA with group membership, song treatment, treatment order (1–3) and the interaction between song treatment and treatment order as fixed factors, and individual identity as a random factor to control for repeated measurements. We used two-tailed Tukey honest significant difference (HDS) tests for two-by-two comparisons. The same comparisons were made with PC2 scores. Then, to investigate the effect of the distance between the territories of tested subjects and the territories of neighbours whose songs were used as a stimulus on PC1 scores, we used a Linear Mixed Model (LMM) fitted with Restricted Estimate Maximum Likelihood (RELM, lme function in R v.2.9.0 [16]). In this model, song treatment (aN and dN) was fitted as a random term to control for treatment effects. Residuals were inspected to ensure normality of error.

**Table 1:** Factor loadings of the response measures on the first two principal components.

Statistics and response measures	PC1	PC2
Eigenvalue	1.50	1.05
Percent of variance	56.3	27.7
Duration of movements at less than 10 m from the loudspeaker	**0.966**	−0.184
Time spent at less than 10 m from the loudspeaker	**0.793**	0.346
Total duration of movements	**0.771**	−0.469
Latency to move	−0.308	−**0.857**

Eigenvalues, variances explained and contributions of the response measures to the first (PC1) and second (PC2) principal components for playbacks of S, aN and dN songs. The total variance explained by PC1 and PC2 was 84.0%. Measures that contributed most to the particular compound variables are in bold.

### Propagation experiment

We broadcast, in an open landscape corresponding to skylarks' habitat, five exemplars of a selected song sequence of 19.7 s duration using a 10 W Megavox Pro mega-6000 loudspeaker placed on a tripod at a height of 2 m from the ground and connected to a Marantz PMD 690 digital recorder, at the intensity estimated to be normal for the birds (same intensity as for playbacks). The song was re-recorded at 0.1 m high and 2 m high using a Marantz PMD 690 numeric recorder connected to a Beyerdynamic M69 microphone (frequency response: 50 Hz à 16 kHz ±1 dB) over seven distances (control signal: 1.56 m; propagated signals: 12.5, 25, 50, 100 m (extreme diameter of a territory), 200 m and 400 m). We analyzed the re-recorded sounds by comparing the propagated signals with the control. Re-recorded signals were digitally filtered (pass-band: 1.6 Hz–7 Hz). We measured signal envelopes to assess modifications of main amplitude fluctuations, signal spectrums to assess modifications of frequency composition, and signal spectrograms to assess modifications of frequency modulation. Each of these measures were averaged (*n* = 5 exemplars) for each propagation distances. Mean envelopes and mean spectrums of control signals were correlated (*r*-values) with those of propagated signals using Bravais-Pearson product-moment. Mean spectrograms were compared using the digital spectrographic cross-correlation method [17] with Avisoft-Correlator v.2.0 [18]. At distances greater than 200 m, we were unable to carry out correlations, the songs being indistinguishable from background noise.

### Song analysis

We selected seven individuals tested during the playback experiments and whose songs had been recorded for the song analysis. The repertoires of syllables and sequences of 3 to 10 syllables of each individual and the repertoires of the adjacent neighbours, distant neighbours and strangers whose songs had been broadcast during the playback experiment were analyzed as described in Briefer et al. [10], by selecting 100 s of a continuous good signal to noise ratio song, labelling syllables on a spectrogram (FFT-Length: 1024; Frame: 100%; Bandwidth: 61 Hz; Resolution: 46 Hz, Hamming window) and examining the sequential organization of syllables using a custom Matlab program (The MathWorks, Natick, MA, USA; see [19]). Sharing of syllables and of sequences of each individual with its corresponding adjacent neighbour, distant neighbour and stranger were then estimated by calculating coefficients of repertoire similarity (RS) as follows: RS = Z/((X+Y) −Z), with X and Y being the total number of syllables or sequences produced by males x and y, and Z being the number of syllables or sequences shared by males x and y [20]. RS values range from 0 to 1, with 1 being maximum sharing. Conventional parametric and non-parametric tests are not suitable for analyses in which each individual is included several times in the different pair-wise comparisons [21], [22]. Thus, we compared RS values calculated between males tested during the playback experiment and their adjacent neighbours, distant neighbours and strangers with two-tailed exact paired permutation tests using the Monte Carlo method. Additionally, we used a permuted correlation test (1000 permutations) for vectors of numeric values containing distances or similarities to test the correlation between RS values calculated between pairs of neighbours (adjacent and distant) and geographical distances between their territories (equivalent of a Mantel test [23]; see also [21]).

Statistical analyses were carried out using R v.2.9.0 [16]. Results attained significance when p<0.05. All means are given with SEs.

## Results

### Playback experiment

**Figure 3:**
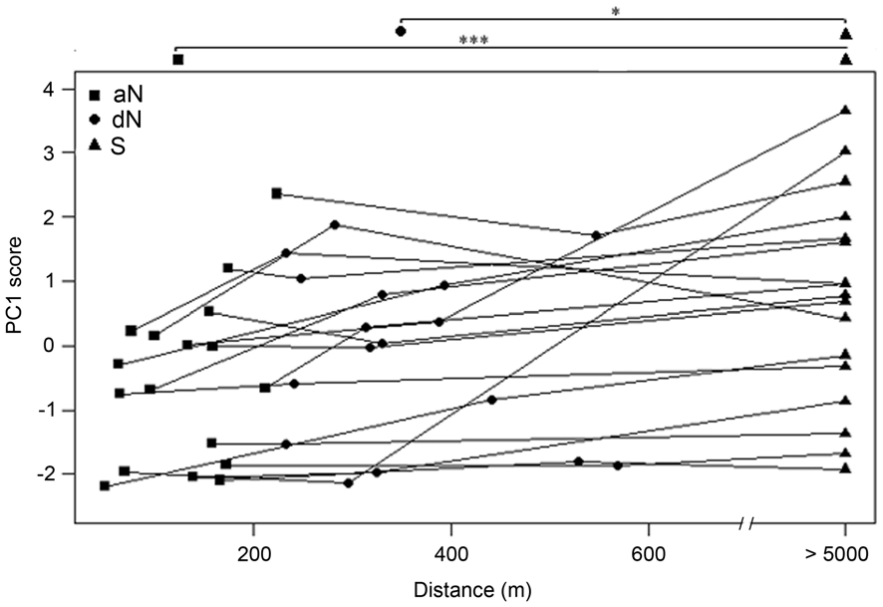
Playback responses. Individual PC1 scores (*n = *17) for playbacks of adjacent Neighbour songs (aN, squares), distant Neighbour songs (dN, circles) and Stranger songs (S, triangles) plotted against the distance between the subject's territory and the territory of the individual whose song was used as a stimulus. Lines show repeated measures of the same individual between song treatments. Higher positive PC1 scores correspond to stronger responses. Responses to S songs are significantly stronger than responses to both aN and dN songs (Tukey HSD test, * *p*<0.05, *** *p*<0.001). Responses to aN and to dN songs are not significantly different and are not affected by the distance between territories.

The first principal component (PC1) explained 56.3% of the variance in the responses measured. The duration of movements around the loudspeaker, the time spent around the loudspeaker and the total duration of movements were strongly correlated with PC1 (Table 1). Higher positive PC1 scores corresponded to stronger responses, i.e. subjects spent more time in movement in the immediate vicinity of the loudspeaker and responded faster. A comparison between PC1 scores showed that responses were significantly different depending on the song treatment (Factorial ANOVA: *F_2,29_* = 8.57, *p* = 0.001). There was no significant effect of treatment order and of group membership (Factorial ANOVA: order, *F_1,29_* = 0.51, *p* = 0.48; group, *F_1,13_* = 0.001, *p* = 0.98) and no significant interaction effect between treatment order and song treatment on PC1 scores (Factorial ANOVA: *F_2,29_* = 0.09, *p* = 0.91). Subjects responded significantly more to S songs than to both aN and dN songs (Tukey HSD test: S and aN, *n* = 17, *p* = 0.0009; S and dN, *n* = 17, *p* = 0.031; Fig. 3). On the other side, responses to aN and dN songs were not significantly different (Tukey HSD test: *n* = 17, *p* = 0.36; Fig. 3). The LMM showed that these responses were not affected by the distance between territories of tested subjects and of their neighbours whose songs were used as a stimulus (LMM: *F_1,31_* = 0.54, *p* = 0.47; Fig. 3).

The second principal component (PC2) explained 27.7% of the variance in the responses measured. The latency to move was more strongly correlated with PC2 than other measures (Table 1). We found no significant effect of song treatment on PC2 scores (Factorial ANOVA: *F_2,29_* = 1.00, *p* = 0.38).

### Propagation experiment

The propagation experiment showed that the correlation between the propagated song sequence (envelope, spectrum and spectrogram) and the control one decreased with the propagation distance (Table 2), with weak correlations from 100 m, especially for the envelope (amplitude modulations) and the spectrogram (frequency modulations).

**Table 2:** Propagation results.

		Distance (m)				
	Microphone Height (m)	12.5	25	50	100	200
Envelope	0.1	0.82	0.61	0.56	0.58	0.48
	2.0	0.61	0.67	0.58	0.41	0.40
Spectrum	0.1	0.88	0.89	0.76	0.69	0.67
	2.0	0.90	0.89	0.86	0.75	0.64
Spectrogram	0.1	0.89	0.92	0.79	0.49	0.29
	2.0	0.89	0.80	0.86	0.60	0.57

Correlations (*r*-values, Bravais-Pearson product-moment) between control and propagated envelopes, spectra and spectrograms (digital spectrographic cross-correlation method). Re-recording height and propagation distances are indicated. All the correlations are significant at *p*<0.001.

### Song analysis

**Figure 4:**
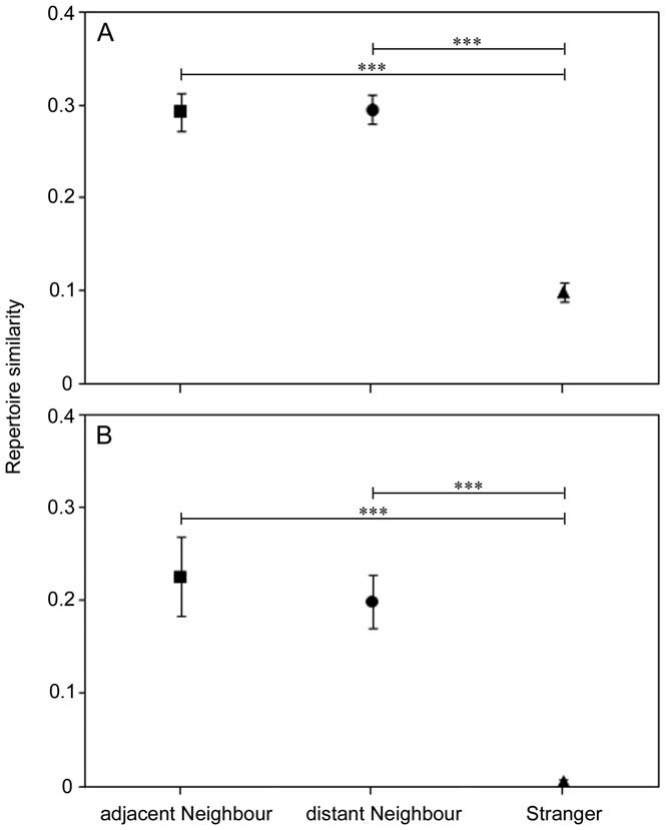
Syllable and sequence sharing. Mean ± SE (*n = *7 pairs for each category) syllable repertoire sharing (A graph) and sequence of 3 to 10 syllables repertoire sharing (B graph) between adjacent neighbours (squares), distant neighbours (circles) and strangers (triangles). Adjacent and distant neighbours share a similar amount of syllables and sequences in their songs and more syllables and sequences than strangers (Exact paired permutation test using the Monte Carlo method, *** *p*<0.001).

RS values calculated between males tested during the playback experiments and their adjacent and distant neighbours were similar (Exact paired permutation test using Monte Carlo method, *n* = 7 RS values between adjacent neighbours and 7 RS values between distant neighbours: Syllables, *p* = 1.00; Sequences, *p* = 0.64, Fig. 4). Furthermore, RS values calculated between neighbours (adjacent or distant) were not significantly correlated with the distance between their territories (Permuted Pearson correlation test, *n* = 14 RS values between neighbours: Syllables, *r = *−0.25, *p* = 0.19; Sequences, *r = *−0.31, *p* = 0.12). RS values calculated between tested males and both their adjacent and distant neighbours were significantly higher than RS values calculated between tested males and strangers (Exact paired permutation test using Monte Carlo method, *n* = 7 RS values between adjacent neighbours, 7 RS values between distant neighbours and 7 RS values between strangers: *p*<0.001 for each comparison (syllables and sequences, adjacent neighbours versus strangers and distant neighbours versus strangers), Fig. 4). Therefore, adjacent and distant neighbours share a similar amount of syllables and sequences in their songs. In contrast, individuals share only few syllables and no sequences with strangers.

## Discussion

We investigated responses of territory owners to songs of their neighbours settled a few territories away in the skylark, a species showing dear-enemy relationships between adjacent neighbours. Our first hypothesis was that a male's response to a given song depends on his familiarity with the singer, in which case his response to a song would increase with the distance to the bird singing this song. The alternative hypothesis was that a male's response to a given song depends on the presence in this song of familiar song components shared by members of the neighbourhood. In this case, we predicted that his response to a neighbour song would not be related to the distance to the territory of the bird singing this song, inducing a similar response to adjacent and distant neighbour songs. Our results show that males respond less aggressively to adjacent and distant neighbours than to strangers. Responses to adjacent and distant neighbour songs were not significantly different and not related to the distance to the territory of the neighbour, thus lending support to our second hypothesis. This indicates that skylark males are tolerant towards every group member, even distant ones whose songs are unlikely to be decoded or even detected as revealed by our propagation results. Because of these dear-enemy-like relationships, members of a group benefit from a reduction in energy and in time spent deterring non-threatening individuals [2].

We cannot rule out that males may perform excursions outside their territories to occasionally approach their distant neighbours so that they would be sufficiently close to experience their songs. However, this is unlikely because, during the breeding season, males remain within their territory to breed and forage at short distances from their nest [24]. We thus believe that a male's response to a given song most probably depends on his familiarity with particular song components in this song that are shared by all group members. Indeed, in the present study, we showed that male skylarks share a similar amount of song components (syllables and sequences of syllables) with their adjacent neighbours and with distant ones. Furthermore, within a group, repertoire sharing is not correlated with the distance between territories (see also [10]). In a previous study, we showed that sequences of syllable shared by males of a given group constitute a group signature used by birds for neighbour-stranger discrimination [8]. Responses of subjects to distant neighbour songs can be compared to responses to chimeric songs (stranger songs in which the group signature was artificially inserted) tested in this previous study. Such chimeric songs elicited significantly less aggressive responses than stranger songs and similar responses as adjacent neighbour songs. These findings indicate that group signatures (microdialects) act as ‘passwords’ allowing territory owners to identify distant neighbours as group members and to show reduced territorial responses towards them. Thus, male skylarks unfamiliar with each other but familiar with a common code display dear-enemy-like relationships. These relationships are not established through experience with the singer as common dear-enemy relationships between adjacent neighbours [2], but through experience with the local microdialect.

Relying on sequence sharing to extend dear-enemies-like relationships to non-adjacent territory owners has not been observed in other bird species, where males usually react strongly to non-adjacent neighbours sharing the same dialect (e.g. song sparrow *Melospiza melodia* [7], ortolan bunting *Emberiza hortulana* [25]). This process could arise in our studied species because, contrary to a vast majority of songbirds living in more continuous habitats, skylark populations have a particularly fragmented distribution, mainly due to human activities, leading to distinct small patches of acoustic variability [10]. A different relationship between distant neighbours may be found in populations of skylarks living in continuous habitats not subjected to high anthropization, where both syllable and sequence sharing are lower between neighbours and decrease with the distance between birds without distinct microdialects [10]. Further studies would be needed in other bird species showing similar group signatures as skylarks (e.g. [26]–[29]) to investigate if the group recognition process identified in our study is widespread in other songbird populations living in fragmented habitats.

To distinguish among neighbours and strangers or among different neighbours, several cues have been suggested to be used by songbirds [3]: (1) repertoire composition (phonology); (2) order of production of repertoire components (syllable types or song types, *i.e.* syntax); (3) distinctive ‘voice’ characteristics and/or subtle differences in the song types versions of each individual [30], [31]. (1) and (2) are probably more important in species with moderate or large repertoires that produce their repertoire components with immediate variety (no repetitions of repertoire components), like skylarks [10] (e.g. European robin *Erithacus rubecula* [32]), because the entire repertoire is produced within a short time interval. On the other side, (3) may be more widespread in species with small repertoires and/or high song sharing (e.g. song sparrow [7]), or in species with very large repertoire and no or eventual variety of repertoire component production (repetitions of repertoire components, e.g. tropical mockingbirds *Mimus gilvus* [33]). Indeed, in such large repertoire species, (1) or (2) would probably require considerable time and neuronal resources to sample and remember the entire repertoire of neighbours (e.g. 6 h to sample most of the syllable repertoire of a given male in tropical mockingbirds [34]). In a previous study on individual recognition [35], we did not find any evidence for voice characteristics in skylarks, *i.e.* the within-individual variation of song frequency and temporal parameters measured was greater than the between-individual variation. However, we found individually distinctive syllables and sequences of syllables that males could potentially use to individually identify their close neighbours. Skylarks seem thus to rely on the composition of the syllable and sequence repertoires rather than on other acoustic cues to differentiate group members from strangers and to individually recognize their adjacent neighbours.

Our playback procedure of distant neighbour song mimicked a group member leaving its territory and attempting to come up closer to the tested male. In this situation, we observed a trend towards a stronger response to the distant neighbour song than to the adjacent neighbour song that a larger sample size might be able to detect (Fig. 3). This trend could be the result of an adjacent-distant neighbour discrimination. We showed previously that males spatially categorized adjacent neighbour songs ([35], see also [36], and [32], [37] for other songbird species with similar repertoire size as the skylark). Here, the birds could have been confused not to hear the “correct” individual neighbour song at the “correct” distance, inducing a slightly stronger response to this displaced group member song.

To conclude, we showed that, in skylarks, even distant neighbours can be identified as group members and considered to be dear-enemies. They are probably discriminated from strangers using the group signature in their song. Such signature may thus act as a key that strengthens group cohesion through a reduction in territorial aggression between group members. Similar studies in other cluster living territorial songbirds could add to our understanding of interactions occurring between and within communication networks.

## References

[1] Mundinger PC, Kroodsma DE, Miller, EH (1982). Microgeographic and macrogeographic variation in the acquired vocalizations of birds.. Acoustic communication in birds.

[2] Temeles EJ (1994). The role of neighbours in territorial systems: when are they ‘dear enemies’?. Anim Behav.

[3] Stoddard PK, Kroodsma DE, Miller, EH (1996). Vocal recognition of neighbors by territorial Passerines.. Ecology and Evolution of Acoustic Communication in Birds.

[4] Naguib M, McGregor PK (2005). Singing interactions in songbirds: implications for social relations and territorial settlement.. Animal Communication Networks.

[5] Gentner TQ, Hulse SH (2000). Perceptual classification based on the component of song in European starlings.. J Acoust Soc Am.

[6] Gentner TQ, Hulse SH, Bentley GE, Ball GF (2000). Individual vocal recognition effect of partial lesions to HVc on discrimination, learning, and categorization conspecific song in adult songbirds.. J Neurobiol.

[7] Wilson PL, Vehrencamp SL (2001). A test of the deceptive mimicry hypothesis in song-sharing song sparrows.. Anim Behav.

[8] Briefer E, Aubin T, Lehongre K, Rybak F (2008). How to identify dear-enemies: the group signature in the complex song of the skylark *Alauda arvensis*.. J Exp Biol.

[9] Aubin T, Brémond JC (1983). The process of species-specific song recognition in the skylark *Alauda arvensis*. An experimental study by means of synthesis.. Z Tierpsychol.

[10] Briefer E, Osiejuk T, Rybak F, Aubin T (2010). Are bird song complexity and song sharing shaped by habitat structure? An information theory and statistical approach.. J Theor Biol.

[11] Briefer E, Rybak F, Aubin T (2008). When to be a dear-enemy: flexible acoustic relationships of neighbouring skylarks *Alauda arvensis*.. Anim Behav.

[12] Specht R (2004).

[13] Craig C (2000).

[14] Sueur J, Aubin T, Simonis C (2008). Seewave: a free modular tool for sound analysis and synthesis.. Bioacoustics.

[15] McGregor PK, McGregor PK (1992). Playback and studies of Animal Communication..

[16] R Development Core Team (2009). R Foundation for Statistical Computing, Vienna, Austria.. http://www.R-project.org.

[17] Khanna H, Gaunt LL, McCallum DA (1997). Digital spectrographic crosscorrelation: tests of sensitivity.. Bioacoustics.

[18] Specht R (2001).

[19] Lehongre K, Aubin T, Robin S, Del Negro C (2008). Individual signature in canary songs: contribution of multiple levels of song structure.. Ethology.

[20] Hultsch H, Todt D (1981). Repertoire sharing and song post distance in nightingales.. Behav Ecol Sociobiol.

[21] Sokal RR, Rohlf FJ, Freeman WH (1995). Biometry∷ the principles and practice of statistics in biological research. 3rd ed..

[22] Mundry R (1999). Testing related samples with missing values: a permutation approach.. Anim Behav.

[23] Mantel N (1967). The detection of disease clustering and a generalized regression approach.. Cancer Res.

[24] Donald PF (2004). Song and song flight..

[25] Skierczynski M, Osiejuk T (2010). Sharing songs within a local dialect does not hinder neighbour-stranger discrimination in ortolan bunting (*Emberiza hortulana*) males.. Behaviour.

[26] Mammen DL, Nowicki S (1981). Individual differences and within-flock convergence in chickadee calls.. Behav Ecol Sociobiol.

[27] Hausberger M, Snowdon CT, Hausberger M (1997). Social influences on song acquisition and sharing in the European starling (*Sturnus vulgaris*).. Social Influences on Vocal Development.

[28] Baker MC (2004). The Chorus Song of Cooperatively Breeding Laughing Kookaburras (Coraciiformes, Halcyonidae: *Dacelo novaeguineae*): Characterization and Comparison Among Groups.. Ethology.

[29] Radford AN (2005). Group-specific vocal signatures and neighbour-stranger discrimination in the cooperatively breeding green woodhoopoe.. Anim Behav.

[30] Lambrechts MM, Dhondt AA (1995). Individual voice discrimination in birds.. Curr Ornithol.

[31] Weary DM, Krebs JR (1992). Great tits classify songs by individual voice characteristics.. Anim Behav.

[32] Brindley EL (1991). Response of European robins to playback of song: neighbor recognition and overlapping.. Anim Behav.

[33] Botero CA, Riveros JM, Vehrencamp SL (2007). Relative threat and recognition ability in the responses of tropical mockingbirds to song playback.. Anim Behav.

[34] Botero CA, Mudge AE, Koltz AM, Hochachka WM, Vehrencamp SL (2008). How reliable are the methods for estimating repertoire size?. Ethology.

[35] Briefer E, Aubin T, Rybak F (2009). Response to displaced neighbours in a territorial songbird with a large repertoire.. Naturwissenschaften.

[36] Stoddard PK, Beecher MD, Horning CL, Campbell SE (1991). Recognition of individual neighbors by song in the song sparrow, a species with song repertoires.. Behav Ecol Sociobiol.

[37] Naguib M, Todt D (1998). Recognition of neighbors song in a species with large and complex song repertoires-the thrush nightingale.. J Avian Biol.

